# Effects of precipitation changes on switchgrass photosynthesis, growth, and biomass: A mesocosm experiment

**DOI:** 10.1371/journal.pone.0192555

**Published:** 2018-02-08

**Authors:** Dafeng Hui, Chih-Li Yu, Qi Deng, E. Kudjo Dzantor, Suping Zhou, Sam Dennis, Roger Sauve, Terrance L. Johnson, Philip A. Fay, Weijun Shen, Yiqi Luo

**Affiliations:** 1 Department of Biological Sciences, Tennessee State University, Nashville, Tennessee, United States of America; 2 Key Laboratory of Vegetation Restoration and Management, South China Botanical Garden, the Chinese Academy of Sciences, Guangzhou, China; 3 Department of Agricultural and Environmental Sciences, Tennessee State University, Nashville, Tennessee, United States of America; 4 Grassland Soil and Water Research Laboratory, United State Department of Agriculture, Temple, Texas, United States of America; 5 Department of Biological Sciences, Northern Arizona University, Flagstaff, Arizona, United States of America; Pacific Northwest National Laboratory, UNITED STATES

## Abstract

Climate changes, including chronic changes in precipitation amounts, will influence plant physiology and growth. However, such precipitation effects on switchgrass, a major bioenergy crop, have not been well investigated. We conducted a two-year precipitation simulation experiment using large pots (95 L) in an environmentally controlled greenhouse in Nashville, TN. Five precipitation treatments (ambient precipitation, and -50%, -33%, +33%, and +50% of ambient) were applied in a randomized complete block design with lowland "Alamo" switchgrass plants one year after they were established from tillers. The growing season progression of leaf physiology, tiller number, height, and aboveground biomass were determined each growing season. Precipitation treatments significantly affected leaf physiology, growth, and aboveground biomass. The photosynthetic rates in the wet (+50% and +33%) treatments were significantly enhanced by 15.9% and 8.1%, respectively, than the ambient treatment. Both leaf biomass and plant height were largely increased, resulting in dramatically increases in aboveground biomass by 56.5% and 49.6% in the +50% and +33% treatments, respectively. Compared to the ambient treatment, the drought (-33% and -50%) treatments did not influence leaf physiology, but the -50% treatment significantly reduced leaf biomass by 37.8%, plant height by 16.3%, and aboveground biomass by 38.9%. This study demonstrated that while switchgrass in general is a drought tolerant grass, severe drought significantly reduces Alamo’s growth and biomass, and that high precipitation stimulates its photosynthesis and growth.

## Introduction

Due to fossil fuel combustion and land-use change, global climate change has been accelerating over the past decades [1]. Global land surface temperature is expected to increase 1.1–6.4°C by the end of the century. The increase in temperature affects the hydrological cycle, and causes more extreme precipitation events [2]. For example, Easterling et al. [3] reported that intensity of precipitation during the growing seasons could increase, resulting in more droughts and flooding in the United States (US). Intense droughts and excessive flooding in California are projected to increase by at least 50% by the end of the 21^st^ century [4]. Changes in precipitation intensities will alter soil water availability and influence ecosystem productivity and biomass [5–11].

To reduce both fossil fuel dependence and greenhouse gas emissions, bioenergy/biofuel crops are promoted as part of the solutions [12–14]. The U.S. Energy Independence and Security Act (EISA) of 2007 mandates at least 36 million gallons of biofuel production a year to displace gasoline by 2022. The U.S. Department of Energy estimated that 907 million Mg of biomass are needed annually to replace 30% of the 2004 petroleum consumption in the US by 2030 [15]. Since cellulosic biofuel crops often grow in less productive soils, and require few inputs of water, fertilizer, and pesticides, the demand of cellulosic biofuels such as switchgrass is increasing [16–19].

Switchgrass (*Panicum virgatum* L.) is a perennial C_4_ grass widely distributed from southern Canada to the US and Mexico. It is one of the most dominant grass species within the tallgrass prairie ecosystem [20,21]. The characteristics of switchgrass that make it more attractable than other grass species include the production of high biomass, high nutrient use efficiencies, tolerant of a broad range of environmental conditions, and ability to sequester atmospheric carbon in the soil [18,22,23]. For example, Schmer et al. [23] reported that the annual switchgrass biomass averaged 5.2–11.1 Mg ha^-1^. Biomass production of switchgrass may be influenced by many factors, such as soil nutrients, varieties, and climatic factors like precipitation [24–27].

Research on switchgrass has been conducted over the past decades, particularly on variety comparisons, field management schemes, nutrient limitation responses, and life cycle assessments [21,28]. Physiological studies have been mostly limited to the study of differences among switchgrass cultivars and different agricultural practices [25, 29–32]. Different switchgrass varieties show different leaf photosynthetic rates, ranging from 25.4 to 35.4 μmol CO_2_ m^-2^s^-1^ [33]. Compared to other crop types, the effects of climate change, such as water stresses on switchgrass, have not been well investigated [21,34,35]. Some studies compared differences in annual precipitation and temperature and found that inter-annual precipitation influences soil water availability and then the physiology of switchgrass [21,29]. The soil water stress significantly reduced switchgrass aboveground biomass, establishment rates of stands, and physiological responses [36–39]. But few experimental studies have been conducted to investigate the responses of switchgrass physiology and growth to climate changes [21,35,39].

Precipitation is a very important factor influencing ecosystem productivity and biomass [5,6,32,34,40–42]. This study was designed to determine the effects of sustained precipitation changes on leaf physiology, growth, and biomass of switchgrass. Specifically, we tested 1) whether there were significant effects of the precipitation treatments on switchgrass physiology, growth, and biomass? and 2) how were switchgrass biomass related to plant physiological and environmental factors?

## Materials and methods

### Experimental facility and design

The experiment was conducted in an environmentally controlled greenhouse at Tennessee State University Agricultural Research and Demonstration Center (Latitude 36.12'N, Longitude 86.89'W, Elevation 127.6 m) in Nashville, Tennessee [43]. Roof panels on the greenhouse opened automatically during clear days and closed during rain. Wall panels also opened automatically to further regulate the temperature, and closed when the temperature was below 20°C. The temperature in the greenhouse was controlled by a Wadsworth Step Up Control system (Arvada, CO). Panels were controlled by a Micro Grow Commercial Greenhouse Control (Temecula, CA) with inputs from rain and wind speed sensors. Temperature varied during the day and night. Light in the greenhouse averaged about 80% of full sunlight.

Switchgrass was grown in large pots (95 L, 50 cm Diameter and 50 cm Height) placed on the greenhouse floor. There were holes in the bottom of the pots to allow free draining. Pots were filled with top soil from an Armour silt loam soil, with pH = 6.2 and low in phosphorus and potassium. No fertilizer was applied during this study. Seeds of “Alamo” switchgrass were planted in a field plot in April 2011, and two-year old switchgrass plants with two to three tillers were transplanted in the large pots in May, 2013. Five plants were planted in each pot with one in the center. Plants were harvested three times during each growing season, at the end of April, July, and October each year. Thus, the whole growing season was separated into three harvest periods (February-April; May-July; August-October).

Five simulated precipitation treatments were applied during the 2014 and 2015 growing seasons in a completely randomized block design with five blocks. Precipitation treatments were defined relative to an ambient precipitation treatment, which applied the annual amount and monthly distribution of rainfall from 1969, which typified the amount and seasonality of precipitation over the past 100 years (1903–2012) for Nashville, TN. Two drought treatments (-33% and -50% of ambient precipitation), and two wet treatments (+33%, and +50% of ambient precipitation) were also used. For the ambient precipitation, monthly precipitation varied from 6.12 cm in May to 15.57 cm in October with a mean monthly precipitation of 9.80 cm (Annual precipitation amount was 1176 cm; S1 Fig). Precipitation treatment applications were automated using a watering timer controller (RSC600i, Raindrip, Inc., Woodland Hills, CA). In 2013, the ambient level treatment was applied to all pots to minimize water stress during establishment. The precipitation treatments began on February 01, 2014. In 2014, pots were watered every three days, three times each day at midnight, in the early morning and later afternoon. Application amounts were adjusted monthly to match monthly variation in precipitation. In 2015, water was added twice each day at midnight and in the early morning, with the same total monthly precipitation amount. In June and July of 2015, we had two incidents where several pots received natural precipitation because the rain sensor failure prevented closing of the roof vents. To compensate for this additional irrigation water, we reduced irrigation in affected pots.

### Measurements

Soil temperature and moisture sensors were buried at 20 cm depth in each pot to continuously monitor soil temperature and moisture using the Watermark Monitor 900M (Irrometer Inc., Riverside, CA). The data were recorded every hour. The soil moisture sensor measures soil matric potential in centibar (cb), which is equal to kilopascal (kPa), over a range of 0 to -239 cb. The larger cb number means the higher soil water content [44].

Maximum leaf photosynthetic rate, stomatal conductance, and transpiration were measured five times during each harvest period using a Li-6400XT Portable Photosynthesis System (Li-Cor, Inc., Lincoln, NE). The fully expanded young leaves of four or five selected tillers in each pot were measured between 10:00am and 3:00pm. Leaf chamber photosynthetic photon flux density was set at 2000 μmol m^-2^s^-1^. Reference CO_2_ was set at ambient CO_2_ concentration in the greenhouse at the measurement (~400 ppm). Temperature was not controlled during the measurements. Measurements were conducted biweekly. Leaves were randomly selected each time for measurements. Instantaneous water use efficiency (WUE_i_) was calculated as a ratio of leaf photosynthesis and transpiration. Leaf temperature was measured at the same time.

The maximum height, average height, and number of tillers in each pot were measured at the end of each harvest period. Maximum height was the measured of the tallest tiller and the average tiller height was measured by averaging five tillers in each pot. Biomass was measured every period after harvesting the aboveground tillers in the pot, dried at 75°C for more than 24 hr to constant mass, and weighed. All plants in the pots were harvested each time. Due to relatively small areas in pots, we did not harvest and measure belowground biomass. Aboveground biomass was reported on a dry basis. Leaf and stem biomass were separately measured and leaf:stem ratio was calculated. Biomass-based WUE (WUE_b_) was calculated as a ratio of aboveground biomass and total water amount applied during each harvest period.

### Statistical analysis

Data analysis was performed using SAS software 9.3 (SAS Inc. Cary, NC) [45]. The effects of precipitation treatment, year, harvest period, and block on soil moisture, soil temperature, leaf physiology, plant height, number of tillers, and aboveground biomass were analyzed using repeated measure analysis of variance (ANOVA). When a significant effect at α = 0.05 level was detected, least significant difference (LSD) was used for multiple comparisons. Regression analysis was conducted to develop the relationships among photosynthesis, transpiration, WUE, number of tillers, tiller height, aboveground biomass, soil temperature, and soil moisture. Bivariate regression was first used to detect the relationships between two variables; then stepwise multiple regression was applied to derive the optimal regression models for physiology, growth, and biomass of switchgrass under all precipitation treatments.

## Results

### Switchgrass physiological variables and growth before the precipitation treatments

In 2013 before the precipitation treatments were applied, there were no significant effects in leaf photosynthesis, stomatal conductance, and tiller growth among treatment plots (S1 Table). Only blocks showed significant effects. We set blocks that paralleled to the greenhouse side wall. The significant block effects indicated that the potential effects of the environmental differences due to pot settings could be partitioned by the block arrangements. Further analyses focused only on the data collected after the precipitation treatments.

### Seasonal variations of soil temperature and moisture among precipitation treatments

The ANOVA test showed that there were significant differences in soil moisture among the precipitation treatments, years and blocks, but no significant difference in soil temperature among the precipitation treatments (Table 1). Soil moisture decreased with the decrease in the amount of water applied. The soil moisture in the +50% treatment was the highest and the -50% precipitation treatments had the lowest soil moisture (Table 2). No difference in soil moisture between the -33% and -50% treatments was detected. Mean soil temperature was about 23°C for all treatments (Table 2).

**Table 1:** Significance of the effects of precipitation treatments, year, their interaction, and block on soil moisture and temperature using ANOVA. Numbers are F values. Stars indicate the level of significance (* = *p*<0.05, ** = *p*<0.01).

Source	Soil Moisture (cb)	Soil Temperature (°C)
**Block**	5.27**	23.55**
**Precipitation**	128.19**	1.33
**Year**	51.74**	5.12*
Precipitation*Year	1.57	0.65

**Table 2:** Multiple comparisons of soil moisture and soil temperature under five precipitation treatments. Same letters indicate no significant difference in a column.

Precipitation Treatment	Soil Moisture (cb)	Soil Temperature (°C)
**+50%**	-81.03±5.26a	23.72±0.30a
**+33%**	-92.90±5.39b	23.78±0.28a
**Ambient**	-149.98±5.78b	24.09±0.29a
**-33%**	-200.12±4.00c	23.95±0.29a
**-50%**	-206.73±4.04c	23.84±0.29a

**Fig 1:**
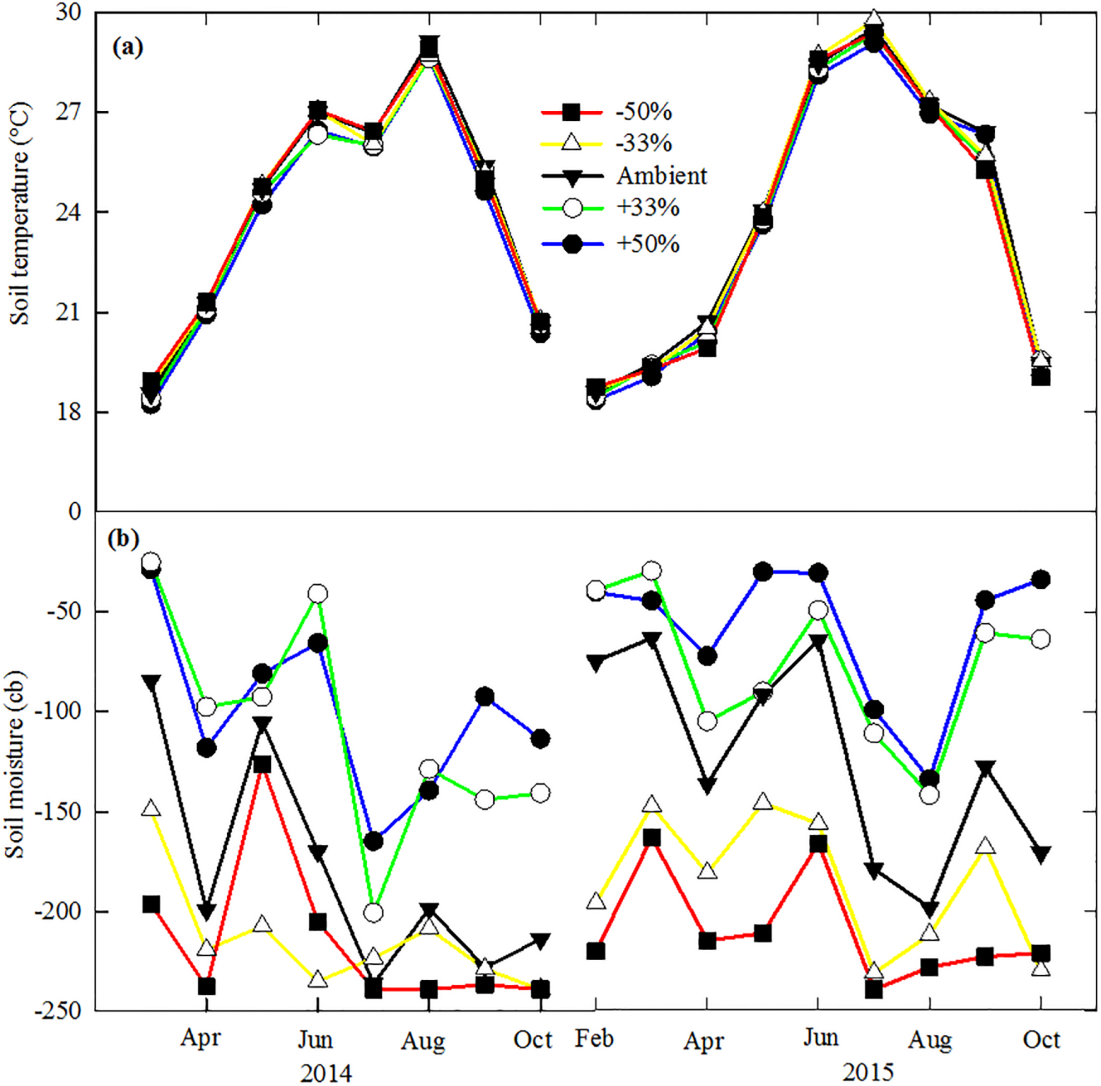
Monthly mean soil temperature and soil moisture in each precipitation treatment from February to October in 2014 and 2015.

Soil temperature showed strong seasonal variations among all treatments in both growing seasons (Fig 1a). The mean monthly soil temperature ranged from 18.6°C to 29.4°C during the growing seasons, with the highest temperature appeared in July or August. Soil moisture also varied seasonally following the precipitation pattern (Fig 1b; S1 Fig), and in the ambient precipitation treatment ranged from -63 to -236 cb during the growing seasons. Soil moistures in the -33% and -50% treatments were below -145 cb, and were mostly above -145 cb in the +33% and +50% treatments.

### Seasonal variations of leaf photosynthesis and transpiration of different precipitation treatments

**Fig 2:**
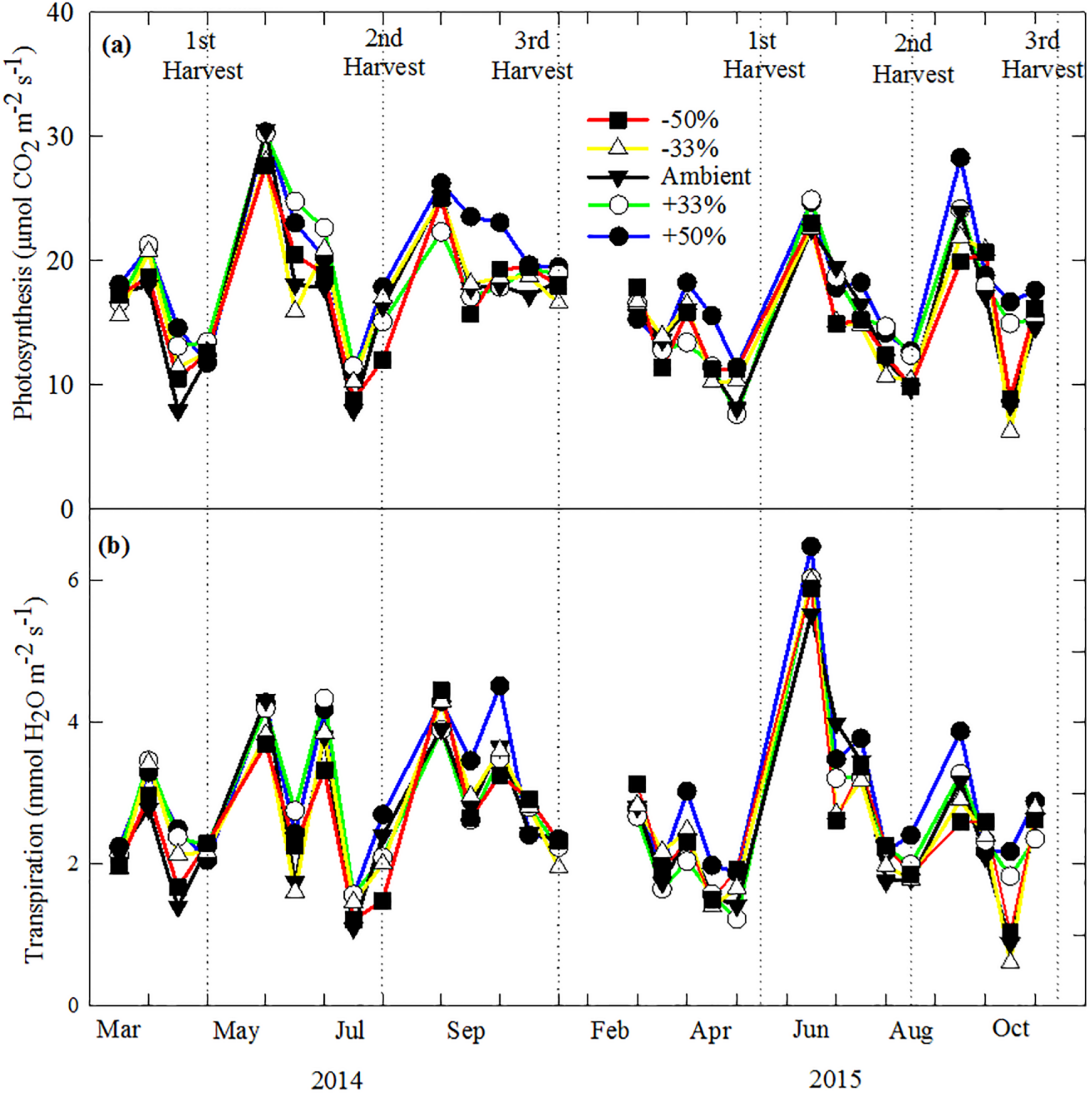
Monthly mean photosynthesis and transpiration in each precipitation treatment from February to October in 2014 and 2015.

The seasonal patterns in leaf photosynthesis and transpiration rates were similar for all treatments. The highest rates occurred in new tillers following each harvest, and rates declined until the next harvest (Fig 2). At each measurement time, the +50% and +30% treatments tended to have high values in leaf photosynthesis and transpiration rates than other treatments. Compared to the ambient precipitation treatment, the +50% treatment enhanced the photosynthesis mostly by 21.3% and transpiration by 22.0%.

### Overall effects of precipitation treatment, harvest period, year, and their interactions on switchgrass physiology, growth, and biomass

Results of ANOVA showed that precipitation treatments, harvest periods, and growing season had significant effects on most of plant physiological variables, plant growth, stem biomass, and aboveground biomass (Table 3). Leaf biomass and leaf:stem ratio were significantly influenced by precipitation treatments, harvest period, but did not change between years. WUE_i_ (a measure of carbon fixed relative to water transpired) did not change among the precipitation treatments, and number of tillers, and height were not different between the two years. The interactive effects of precipitation treatment and harvest period were significant for photosynthesis, stomatal conductance, number of tillers, height, leaf biomass, stem biomass, and aboveground biomass. Significant interactive effects between precipitation treatments and growing season year were found for stomatal conductance.

**Table 3:** Significance of the effects of treatment, harvest period, their interactions, and block on leaf physiology, growth, and biomass using ANOVA in two years.

Source	P_n_	g_s_	E	WUE_i_	WUE_b_	N_tiller_	H_max_	H_mean_	B_above_	B_Leaf_	B_Stem_	LS
**Block**	1.12	2.51*	4.21**	6.97**	10.92**	1.67	22.39**	36.75**	12.48**	7.78**	11.60**	2.91*
**Precipitation**	16.31**	5.32**	8.63**	0.81	2.83*	4.02**	67.57**	65.06**	36.61**	21.58**	32.87**	2.47*
**Year**	138.67**	67.42**	10.71**	42.30**	19**	24.56**	1.04	3.10	7.41**	0.06	8.32**	2.11
**Harvest**	94.32**	93.14**	89.34**	15.29**	117.14**	33.04**	284.52**	235.58**	152.01**	65.02**	190.86**	34.57**
**Precipitation x Harvest**	2.14*	2.81**	0.89	0.37	1.59	3.23*	26.13**	18.94**	3.99**	2.68**	4.13*	1.53
**Precipitation x Year**	0.85	2.58*	1.02	0.70	0.67	0.38	0.56	0.37	0.17	0.45	0.08	0.34

P_n_: Leaf maximum photosynthesis (μmol CO_2_ m^-2^s^-1^); g_s_: Stomatal conductance (mol H_2_O m^-2^s^-1^); E: Transpiration (mmol H_2_O m^-2^s^-1^); WUE_i_: Instantaneous water use efficiency (μmol mmol^-1^); WUE_b_: Biomass-based water use efficiency (g L^-1^); N_tiller_: Number of tillers; H_max_: Maximum plant height (cm); H_mean_: Mean plant height (cm); B_above_: Aboveground biomass (g pot^-1^); B_leaf_: Leaf biomass (g pot^-1^); B_stem_: Stem biomass (g pot^-1^); LS: Leaf and stem biomass ratio. Numbers are F values. Stars indicate the level of significance (* = *p*<0.05, ** = *p*<0.01).

### Effects of precipitation treatment on switchgrass physiology, growth, and biomass

**Fig 3:**
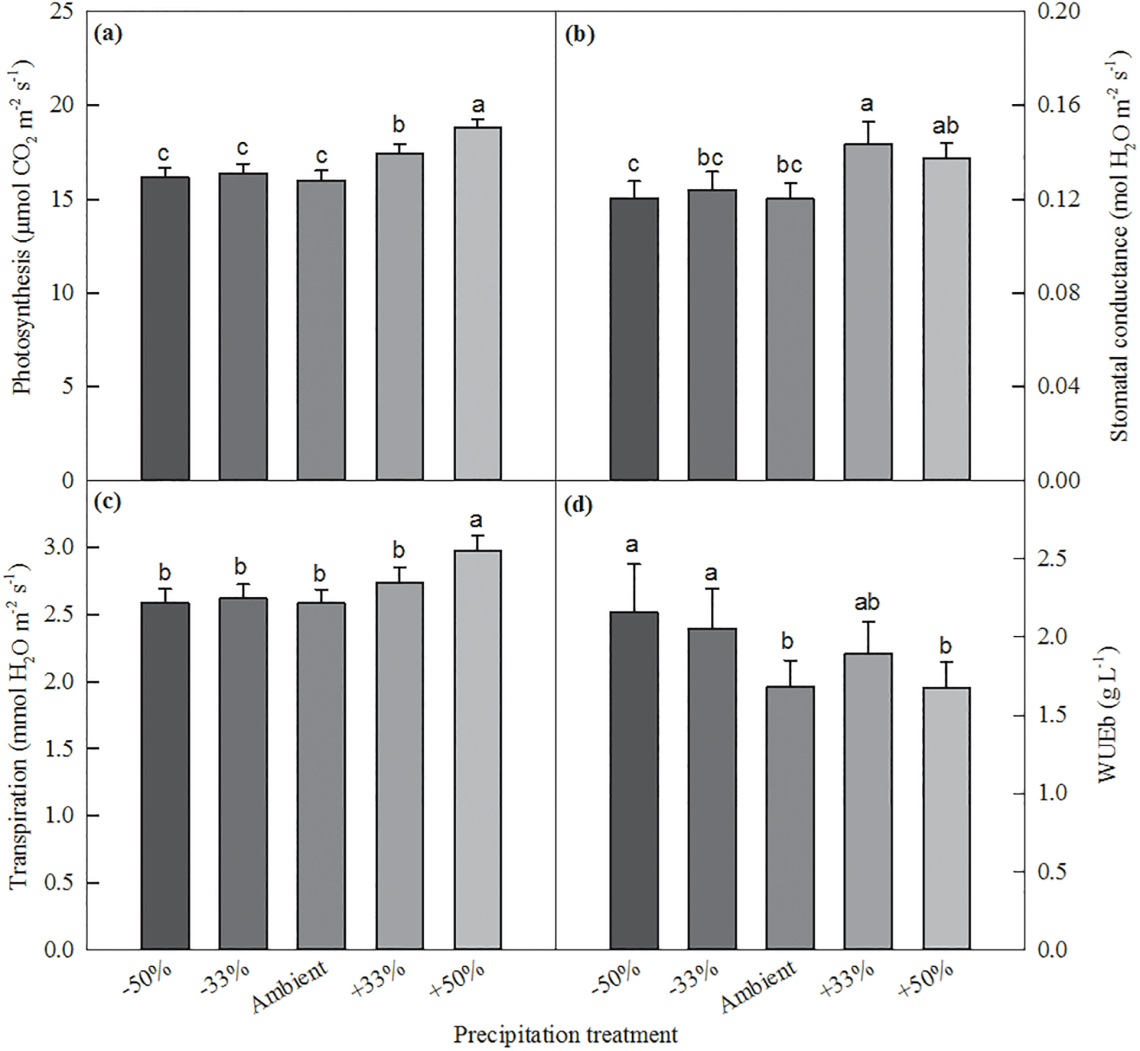
Multiple comparisons of leaf photosynthesis, stomatal conductance, transpiration, and water use efficiency among different precipitation treatments. Differences of variables among treatments labeled with the same letter are not significant at α = 0.05 level.

The precipitation treatment significantly influenced most of the physiological variables measured (Table 3). Among all treatments, the +50% treatment had the highest photosynthesis rate (18.74 μmol CO_2_ m^-2^s^-1^) (Fig 3a), 15.9% higher than the ambient treatment. The +33% treatment had lower photosynthesis than the +50% treatment, but was 8.1% higher than the ambient treatment. No difference in photosynthesis was found between the ambient precipitation and two drought treatments. For stomatal conductance, the +33% treatment had significant higher value than the -50 treatment, but there was no difference among other three treatments (Fig 3b). Similar response pattern of transpiration was found as photosynthesis (Fig 3c). As a result, WUE_i_ wasn’t influenced by the precipitation treatment. WUE_b_ was slightly higher in the drought treatments than the ambient precipitation (Fig 3d). The wet treatments did not influence WUE_b_.

**Fig 4:**
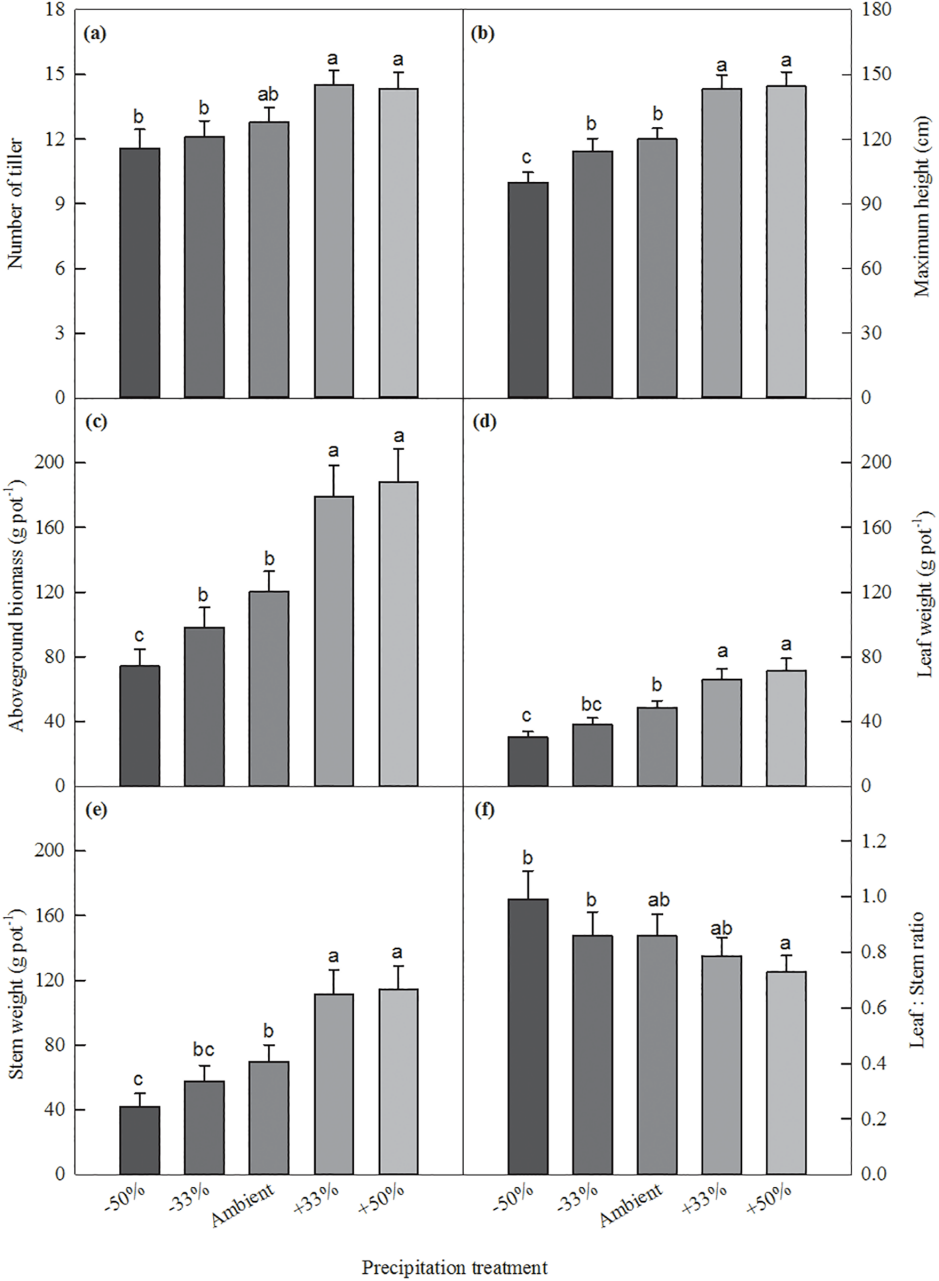
Multiple comparisons of number of tillers, maximum height, and biomass of leaf, stem and total plant, and leaf:stem ratio among different precipitation treatments. Differences of variables among treatments labeled with the same letter are not significant at α = 0.05 level.

Precipitation significantly influenced plant growth and biomass (Table 3). Compared to the ambient precipitation, the wet (+30% and +33%) treatments did not influence the number of tillers produced, but the two drought (-50% and -33%) treatments produced significantly lower number of tillers (Fig 4a). The plants in the wet treatments grew significantly taller (144.2 cm, 20.2%) than the ambient precipitation, and the -50% treatment significantly reduced plant height (100.7 cm, 16.3%). Aboveground biomass was increased by 56.7% to 185.8 g pot^-1^ in the +50% treatment, by 49.6% in the +33% treatment, and reduced by 38.9% to 72.5 g pot^-1^ in the -50% treatment, compared to the ambient precipitation (118.6 g pot^-1^; Fig 4c). Both leaf and stem biomass were increased in the wet treatments, and the severe drought -50% treatment significantly reduced the leaf and stem biomass (Fig 4d and 4e). Leaf biomass and stem biomass were increased by 46.6% and 63.8% in the +50% treatment, and reduced by 37.8% and 39.9% in the -50% treatment, respectively. Leaf:stem ratio in the drought treatments was significantly higher than the +50 treatment (Fig 4f). But no difference in leaf:stem ratio was found between the ambient precipitation with either the drought or wet treatment.

### Effects of harvest period on switchgrass physiology, plant growth, and biomass

Significant differences for leaf physiology, growth, and biomass were observed among the three harvest periods (Table 4). The maximum leaf photosynthesis, stomatal conductance and WUE_i_ were higher in the 3^rd^ harvest period (August-November), but height of plants were significantly higher in the 1^st^ (February-April) and 2^nd^ (May-July) harvests. The highest leaf transpiration and WUE_b_ appeared during the 2^nd^ harvest period. Leaf biomass, stem biomass, and aboveground biomass were higher in the 2^nd^ harvest period, and much lower in the 3^rd^ harvest. Leaf:stem ratio was lower in the 2^nd^ harvest period than other two periods.

**Table 4:** Multiple comparisons of leaf physiology, growth, and biomass of switchgrass among three different harvest periods.

Harvest Period	P_n_	g_s_	E	WUE_i_	WUE_b_	N_tiller_	H_max_	H_mean_	B_above_	B_Leaf_	B_Stem_	LS
**Feb-Apr**	14.61c	0.093c	2.27c	6.67b	1.91b	9.53b	140.0a	85.58a	125.30b	59.30b	66.94b	0.97a
**May-Jul**	17.78b	0.132b	3.03a	6.59b	2.98a	14.49a	144.4a	84.52a	213.98a	70.31a	146.45a	0.54b
**Aug-Oct**	18.95a	0.165a	2.83b	7.25a	0.78c	15.00a	90.2b	49.27b	56.71c	24.66c	26.41c	1.03a

P_n_: Leaf maximum photosynthesis (μmol CO_2_ m^-2^s^-1^); g_s_: Stomatal conductance (mol H_2_O m^-2^s^-1^); E: Transpiration (mmol H_2_O m^-2^s^-1^); WUE_i_: Instantaneous water use efficiency (μmol mmol^-1^); WUE_b_: Biomass-based water use efficiency (g L^-1^); N_tiller_: Number of tillers; H_max_: Maximum plant height (cm); H_mean_: Mean plant height (cm); B_above_: Aboveground biomass (g pot^-1^); B_leaf_: Leaf biomass (g pot^-1^); B_stem_: Stem biomass (g pot^-1^); LS: Leaf and stem biomass ratio. Means followed by the same letter in a column are not significantly different at the α = 0.05 level.

### Variations in switchgrass physiology, plant growth and biomass between the two years

All variables measured showed significant differences between two growing seasons. Leaf photosynthesis, transpiration, WUE_i_ and WUE_b_ were higher in 2014 than in 2015 (Table 5). Plant heights and leaf:stem ratio were similar in two years. The aboveground biomass, leaf and stem biomass in 2014 were also significantly higher than these in 2015.

**Table 5:** Multiple comparisons of leaf physiology, growth and biomass of switchgrass.

Year	P_n_	g_s_	E	WUE_i_	WUE_b_	N_tiller_	H_max_	H_mean_	B_above_	B_Leaf_	B_stem_	LS
**2014**	18.42a	0.144a	2.80a	7.08a	2.15a	11.52a	125.42a	74.10a	142.05a	54.50a	86.77a	0.81a
**2015**	15.57b	0.112b	2.64b	6.49b	1.64b	14.57b	124.16a	71.98a	121.95b	49.90b	72.64b	0.87a

P_n_: Leaf maximum photosynthesis (μmol CO_2_ m^-2^s^-1^); g_s_: Stomatal conductance (mol H_2_O m^-2^s^-1^); E: Transpiration (mmol H_2_O m^-2^s^-1^); WUE_i_: Instantaneous water use efficiency (μmol mmol^-1^); WUE_b_: Biomass-based water use efficiency (g L^-1^); N_tiller_: Number of tillers; H_max_: Maximum plant height (cm); H_mean_: Mean plant height (cm); B_above_: Aboveground biomass (g pot^-1^); B_leaf_: Leaf biomass (g pot^-1^); B_stem_: Stem biomass (g pot^-1^); LS: Leaf and stem biomass ratio. Means followed by the same letter in a column are not significantly different at the α = 0.05 level.

### Relationships among aboveground biomass, leaf physiological variables, soil temperature, and soil moisture of different precipitation treatments

**Fig 5:**
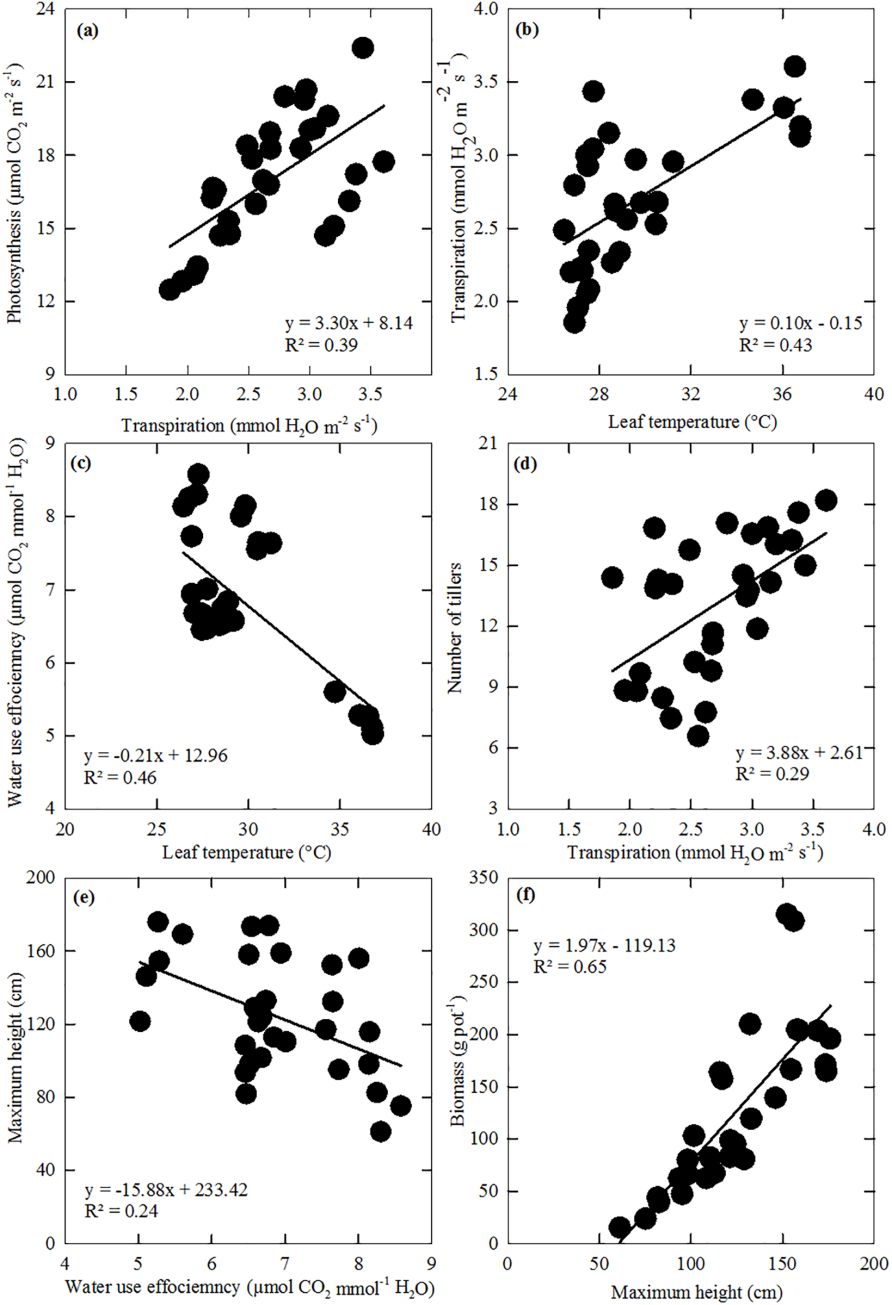
Relationships among leaf photosynthesis, transpiration, water use efficiency (WUE_i_), number of tillers, plant height, biomass, leaf temperature, soil temperature, and soil moisture under all precipitation treatments. Sample size n = 30. All models are significant at α = 0.05 level.

Bivariate regression results showed that leaf photosynthesis was significantly correlated with transpiration (Fig 5a), similar to previous studies [21,46,47]. Transpiration increased with leaf temperature (Fig 5b) and WUE_i_ was negatively correlated to transpiration (Fig 5c). Tiller number was positively influenced by transpiration (Fig 5d), but height was negatively related to WUE_i_ (Fig 5e). A strong positive relationship was found between biomass and plant height (Fig 5f).

Multiple regression analysis showed that leaf photosynthesis was correlated to transpiration, positively related to soil moisture, but negatively related to leaf temperature (Table 6). Transpiration was correlated to photosynthesis, and positively related to leaf temperature. WUE_i_ was negatively related to leaf temperature and positively related to soil temperature. The number of tillers increased with WUE_i_ and soil moisture. Plant height was positively related to soil moisture, but negatively to number of tillers and WUE_i_. Aboveground biomass was positively influenced by WUE_i_, plant height, and soil temperature, but negatively influenced by leaf temperature.

**Table 6:** Relationships of leaf physiological variables, growth, and biomass with soil temperature, soil moisture, and other variables.

Response variable	Model	R^2^
**Photosynthesis**	P_n_ = 17.60+4.98E-0.72Tl+0.29T_s_	0.81*
**Transpiration**	E = -2.36+0.12Pn+0.10Tl	0.86**
**WUE**_**i**_	WUE_i_ = 12.61–0.28Tl+0.11T_s_	0.54**
**Number of tillers**	N_tiller_ = 4.53+4.21E+0.018M	0.42**
**Maximum Height**	H_max_ = 374.6+0.30M -23.59WUE_i_-3.20N_tiller_	0.59**
**Aboveground Biomass**	B_above_ = -563.20+0.18M+24.93WUE_i_+10.43T_s_+2.33H_max_	0.95**

P_n_: Leaf maximum photosynthesis (μmol CO_2_ m^-2^s^-1^); E: transpiration (mmol H_2_O m^-2^s^-1^); WUE_i_: water use efficiency (μmol mmol^-1^); N_tiller_: number of tillers; H_max_: maximum plant height (cm); B_above_: Aboveground biomass (g pot^-1^); T_l_: leaf temperature (°C); T_s_: soil temperature (°C); M: soil moisture (cb); R^2^: coefficient of determination. Stars indicate the level of significance (* = *p*<0.05, ** = *p*<0.01).

## Discussion

The primary objective of this study was to understand the physiological variables and growth responses of switchgrass to precipitation changes. We found that increased precipitations significantly enhanced plant aboveground biomass by stimulating leaf photosynthesis, but mostly, by increasing leaf biomass, stem biomass and height, compared to the ambient precipitation. The significantly reduced aboveground biomass in the severe drought (-50%) treatment was caused by the reduced growth of leaf and stem, not by leaf photosynthesis changes. Among all precipitation treatments, leaf photosynthesis and transpiration were significantly correlated and influenced by temperature. Plant growth and biomass were influenced by soil moisture and water use efficiency. This study provided direct evidence that sustained precipitation changes could have significant impacts on switchgrass physiology and growth, and severe drought would significantly reduce switchgrass growth and biomass. It is worth noting that biomass yield of switchgrass Alamo could be negatively affected by water logging due to heavy precipitation in poorly drained fields [48,49].

In this study, we found that drought treatments reduced plant growth and biomass compared to the ambient precipitation treatment, but did not change much of leaf photosynthesis and transpiration rates, particularly under the -33% treatment (Fig 3). The reduced growth and biomass of switchgrass under the drought treatments have been reported in previous studies. For example, Sanderson & Reed [29] and Hartman et al. [21] found that switchgrass tiller height, tiller number, and aboveground biomass were reduced by water deficits. Barney et al. [50] also reported that drought treatments reduce tiller number, leaf area, and biomass production by up to 80%. Wang et al. [51] found that leaf photosynthesis was not influenced by drought treatment when leaf water potential was larger than -1 Mpa, but decreased with decreasing leaf water potential when it was lower than -1 Mpa. In this study, we did not find significant differences in tiller number between the drought and ambient precipitation treatments, but leaf biomass, stem biomass, and height of switchgrass were significantly reduced in the severe drought treatment.

Lower levels of precipitation amounts and soil moisture contents often limit the stomatal conductance leading to the photosynthesis rate decreases, and finally decrease plant height and biomass [30]. However, we did not find any significant differences in leaf photosynthesis, transpiration and WUE_i_ between the drought treatments and the ambient precipitation (Fig 3). This seemed to be contradictory to some previous studies, as leaf photosynthetic rate is often reduced under the drought treatments. For example, Barney et al. [50] showed a 50% reduction in switchgrass leaf photosynthetic rate when the soil water potential was below -1.5 MPa. Knapp [52] reported that under severe water stress, switchgrass photosynthesis decreases dramatically. The different responses in this study could be related to our growing conditions and harvests. Temperature in the greenhouse was higher than the outside field and plants had extended growing seasons. During the whole growing seasons, plants were harvested three times. The differences in leaf photosynthesis among precipitation treatments tended to be smaller when plants were very young or before harvests. In addition, biomass may have a poor relationship with photosynthetic rate (carbon gain) in drought treatments [21,53]. A recent study of switchgrass genotypes growing under different temperatures showed that variations in leaf-level photosynthesis may not scale up to final biomass yield, as leaf area, leaf architecture, and canopy development could contribute to final biomass yield [54].

The increased precipitation treatments increased photosynthesis, transpiration, maximum tiller height and biomass, compared to the ambient precipitation treatment (Fig 3), similar to some precious studies [55,56]. Plants in the wet treatments had higher photosynthetic rates, grew taller, and produced more biomass compared to those in the ambient precipitation treatment (Table 2). Hartman et al. [21] found that increased precipitation increased leaf photosynthesis and stomatal conductance in early growing seasons, but not in the middle or late seasons. Increased precipitation also stimulated switchgrass growth by producing more tillers and biomass. Abdulahi et al. [8] demonstrated that more frequent irrigation could mostly increase switchgrass biomass. A meta-synthesis of switchgrass yield showed that the annual yield of switchgrass increased with annual precipitation amount [57]. The large increases in aboveground biomass under the wet treatments in this study were mostly caused by the enhanced leaf growth and stem development, with small increases in tiller number. Both plant height, biomass and number of tillers increased with soil moisture, but biomass was more closely related to plant height than number of tillers among all precipitation treatments, indicating that precipitation stimulates more individual tiller growth to access light more than vegetation spread.

WUE measures carbon/biomass produced relative to water consumed [47,58]. WUE_i_ was not influenced by the different precipitation treatments. This is a little surprising, as we expected that WUE_i_ could be enhanced by precipitation increases. We did find that leaf photosynthetic rates were significantly higher under the +33% and +50% treatments, but water uses (transpirations) were also enhanced, as a results, WUE_i_ did not change. Barney et al. [50] reported a similar result. WUE_i_ was 6 μmol mmol^-1^ following a moisture stress period (20% deficit). This value was similar to our results and within the normal range (5.8–6.8 μmol mmol^-1^, [50]). But Hartman et al. [21] reported a higher WUE_i_ in the decreased precipitation treatment compared to the increased precipitation treatment, and WUE_i_ decreased over the growing season with a range of 3.16 to 4.71 μmol mmol^-1^ [59]. They found that switchgrass lowered transpiration rates and stomatal conductance under moisture deficit conditions. In this study, no significant difference in transpiration was found between the drought and the ambient precipitation treatments over the two growing seasons. We found slightly higher biomass-based WUE_b_ under the drought treatments, as less water was applied though the biomass was lower in the drought treatments. It is worth noting that higher WUE is a good property for plant species/varieties, but treatments that increasing WUE may not always contribute to more plant growth or higher biomass, as observed in the drought treatments of this study.

Leaf physiology, plant growth and biomass of switchgrass differed significantly during the three harvest periods and interactively with precipitation treatments. The difference in leaf photosynthetic rate among harvest periods could be related to the changes in leaf temperature. Temperature was lower during the 1^st^ harvest and higher during the 3^rd^ harvest, and resulted in a lower photosynthetic rate during the 1^st^ harvest and a higher photosynthesis during the 3^rd^ harvest period. Within each harvest period, leaf photosynthesis and stomatal conductance declined over time (Fig 2). Similar results were reported by Hartman & Nippert [59] who reported that maximum leaf photosynthesis decreased from 30 to 10 μmol m^-2^ s^-1^ over the course of the growing season. Gao et al. [30] also reported that leaf photosynthesis of switchgrass in arid environments decreased from 17 μmol m^-2^ s^-1^ in May to 8 μmol m^-2^ s^-1^ in September. The declines of photosynthesis could be related to leaf development and soil moisture conditions [60] (Fig 1). Soil moisture often strongly influences the xylem pressure potential to cause the performance change of stomatal and leaf photosynthesis [29]. The last harvest had significantly lower aboveground biomass and plant height, compared to previous harvests, this could be due to higher air temperature and lower soil moisture contents. Lower biomass in late harvest of switchgrass has also been reported in field studies [12,61]. Leaf:stem ratio also varied among three harvests. Similar changes were found at development stages or under different environmental factors [62–64]. For example, Somleva et al. [62] showed that leaf:stem ratio of switchgrass dropped from 0.9–1.1 in vegetative tillers to 0.5–0.7 in tillers at a reproductive stage. In a field study, Tian et al. [63] found that leaf:stem ratio of Alamo decreased from ~1.0 in early vegetative stage to 0.20–0.25 at harvest in October, lower than our results. Changes of leaf:stem ratio at different harvests were mainly due to non-synchronized growths of plant organs, but might also be related to growing temperature [64].

Significant differences in leaf physiology and aboveground biomass were found between the two growing seasons, with the values in 2015 lower than those in 2014. The reasons for this could be due to the difference in temperature and nutrient deficiencies in the soil [65,66]. Air temperature in the greenhouse was set within a temperature range and controlled by the Wadsworth Step Up control system that automatically opened and closed the roofs and window panels. It seems that soil temperature in the greenhouse during the 2015 season was slightly higher than in 2014, particularly from June to August (Fig 1a). High summer temperature might reduce biomass production. In addition, no fertilizer was applied during the 3 years of the experiment. Nutrient limitation might have contributed to the decrease in biomass production in 2015. Further studies are needed to test the interaction of nutrient and precipitation change and whether nutrient is still sufficient for switchgrass growth [29, 67].

## Conclusions

To conclude, we found significant effects of precipitation changes on leaf physiology and plant growth and biomass of switchgrass. Under the reduced precipitation treatments, leaf maximum photosynthetic rate and transpiration were not significantly influenced. But the severe drought treatment significantly reduced leaf biomass, stem biomass, and plant height, suggesting that lower water availability had more influences on leaf and stem development than physiology, and caused the reduced aboveground biomass. To develop high producing switchgrass cultivars for drier environments, attention should be paid to the traits related to leaf initiation and development. While switchgrass can tolerate drought conditions, precipitation increases could significantly enhance leaf photosynthesis, transpiration, and more on leaf and stem growth, and increase aboveground biomass. Thus, adequate irrigation under the drought condition could improve switchgrass growth and biomass. To verify whether the results from this mesocosm study could be applied in the field condition, more field experiments with multiple levels of precipitation treatment with switchgrass need to be conducted.

## Supporting information

S1 Fig Monthly irrigation amount in the ambient precipitation treatment. (DOCX)

S1 Table Significance of the effects of precipitation treatments, harvest period, their interaction, and block on leaf physiology, growth, and biomass using ANOVA before the precipitation treatments (in 2013). Numbers are F values. Stars indicate the level of significance (* = *p*<0.05, ** = *p*<0.01).
(DOCX)
