# Supplementary material for: Effects of precipitation changes on switchgrass photosynthesis, growth, and biomass: A mesocosm experiment
Source: PLoS One. 2018 Feb 8;13(2):e0192555. doi: 10.1371/journal.pone.0192555 (PMC5805322; doi:10.1371/journal.pone.0192555)
Supplement: S1 Fig — (DOCX) [file pone.0192555.s001.docx]

**S1 Fig. Monthly irrigation amount in the ambient precipitation treatment.**
