# Supplementary material for: Effects of precipitation changes on switchgrass photosynthesis, growth, and biomass: A mesocosm experiment
Source: PLoS One. 2018 Feb 8;13(2):e0192555. doi: 10.1371/journal.pone.0192555 (PMC5805322; doi:10.1371/journal.pone.0192555)
Supplement: S1 Table — Numbers are F values. Stars indicate the level of significance (* = p<0.05, ** = p<0.01). (DOCX) [file pone.0192555.s002.docx]

**S1 Table. Significance of the effects of precipitation treatments, harvest period, their interaction, and block on leaf physiology, growth, and biomass using ANOVA before the precipitation treatments (in 2013).** Numbers are F values. Stars indicate the level of significance (^*^=*p*<0.05, ^**^=*p*<0.01).

| Source | Leaf Photosynthesis (μmol CO_2_ m^-2^ s^-1^) | Stomatal Conductance (mol H_2_O m^-2^ s^-1^) | Transpiration (mmol H_2_O m^-2^ s^-1^) | WUE (μmol CO_2_ mmol^-1^ H_2_O) | Number of tillers | Maximum Height (cm) | Average Height (cm) | Above-ground biomass (g pot^-1^) |
| --- | --- | --- | --- | --- | --- | --- | --- | --- |
| Block | 2.47* | 6.76^**^ | 1.15 | 2.10 | 2.11 | 3.84** | 6.10** | 0.81 |
| Precipitation | 0.23 | 0.68 | 0.24 | 2.37 | 1.65 | 0.20 | 0.68 | 0.65 |
| Harvest period | 1623.92** | 123.12** | 1604.10** | 102.18** | -- | -- | -- | -- |
| Precipitation X Harvest period | 0.44 | 0.21 | 0.64 | 2.28* | -- | -- | -- | -- |
